# Identification of unfavourable paired cell interactions in follicular lymphoma using hypothesized interaction distribution (HID) analysis

**DOI:** 10.1186/2051-1426-2-S3-P260

**Published:** 2014-11-06

**Authors:** James Mansfield, Lilli Nelson, Kim Linton, Chris Rose, Richard Byers

**Affiliations:** 1 PerkinElmer, Hopkinton, MA, USA; 2 University of Manchester, Manchester, UK

## Objective

Histopathological prognostication relies on pattern recognition, but as the number of biomarkers increases, human prognostic pattern recognition ability decreases. We have developed an automated quantitative method, hypothesized interaction distribution (HID) analysis, for identifying prognostic patterns of multiple biomarkers in situ. For follicular lymphoma (FL) we have previously demonstrated favourable significance of the transcription factor Yin Yang 1 (YY1) and infiltrating T cells (CD3 +ve) and unfavourable significance of infiltrating macrophages (CD68 +ve).

## Method

To determine whether the pattern of these biomarkers was important we used HID analysis to determine prognostic significance of their pattern. A tissue microarray (n = 53) was used in triplex immunohistochemistry for YY1, CD3 and CD68, and multispectral imaging used to determine the spatial locations of cells either single, double or triple +ve for YY1, CD3 and CD68. Computer analysis was used to identify doublets of cells within a defined distance of each other such as; YY1/YY1, CD68+/CD68+, YY1&CD68+/YY1&CD3+, and so on for all possible combinations (136 in total). From this a co-occurrence matrix was derived summarising the relationships of the cells and used in K-nearest neighbour and Kaplan-Meier survival analyses using leave-one-out cross validation. The co-occurrence matrix can was also expressed visually as a heat-map.

## Results

Two classes with statistically significantly different survival functions (P = 0.025), were identified with median survival of 43 and 112 months. Interestingly, the survival functions appear to be identical until about 30 months, after which they diverge. This indicates that HID analysis may be able to identify patients whose disease will transform. A heat map showed no clear small subset of interactions that appears relate to improved survival, indicating that all the interactions may be important. Linear modelling identified two interactions that may be predictive of survival one of which corresponded to the interaction between pairs of cells positive for the CD3 (P = 0.029), indicating that T cell interaction may be important for survival.

## Conclusion

An automated method for identifying prognostic patterns in multiply stained tissue sections identified two classes with significantly different outcomes. No single subset of interactions drove this difference, though linear modelling showed that higher levels of T cell interaction were associated with a favourable outcome. This is in line with previous studies, though was identified using the HID method automatically without any a priori knowledge and without expert histopathological input. The method can be extended to higher order multiplex analysis, enabling identification of prognostic patterns of multiple biomarkers.

